# Maternal investment and early thermal conditions affect performance and antipredator responses

**DOI:** 10.1093/beheco/arae035

**Published:** 2024-04-26

**Authors:** Maider Iglesias-Carrasco, Jiayu Zhang, Daniel W A Noble

**Affiliations:** Evolution and Ecology of Sexual Interactions Group, Doñana Biological Station-CSIC, Seville, 41092, Spain; Division of Ecology and Evolution, Research School of Biology, The Australian National University, Canberra, Australia; Division of Ecology and Evolution, Research School of Biology, The Australian National University, Canberra, Australia; Division of Ecology and Evolution, Research School of Biology, The Australian National University, Canberra, Australia

**Keywords:** Bayesian multivariate mixed effects models, incubation temperature, *Lampropholis*, morphology, skink, yolk-reduction

## Abstract

Exposure to increased temperatures during early development can lead to phenotypic plasticity in morphology, physiology, and behavior across a range of ectothermic animals. In addition, maternal effects are known to be important contributors to phenotypic variation in offspring. Whether the 2 factors interact to shape offspring morphology and behavior is rarely explored. This is critical because climate change is expected to impact both incubation temperature and maternal stress and resource allocation. Using a fully factorial design, and Bayesian multivariate mixed models, we explored how the manipulation of early thermal environment and yolk-quantity in eggs affected the morphology, performance, and antipredator behavior of 2 sympatric Australian skink species (*Lampropholis delicata* and *L. guichenoti*). We found that juveniles from the hot treatment were larger than those on the cold treatment in *L. guichenoti* but not *L. delicata*. Using repeated behavioral measures for individual lizards, we found an interaction between incubation temperature and maternal investment in performance, with running speed being affected in a species-specific way by the treatment. We predicted that changes in performance should influence antipredator responses. In support of this prediction, we found that maternal investment impacted antipredator behavior, with animals from the yolk-reduced and cold treatment resuming activity faster after a simulated predatory attack in *L. delicata*. However, the prediction was not supported in *L. guichenoti*. Our results highlight the importance of exploring the multifaceted role that environments play across generations to understand how different anthropogenic factors will impact wildlife in the future.

## Teaser

Early thermal conditions can interact with maternal resource investment to shape offspring performance and behavior. While anthropogenic climate change is expected to alter the thermal conditions during development, it is also likely to impact the resource availability and stress levels of mothers. In this study we found that these two factors can interact to shape offspring phenotype, which could have important consequences for wildlife conservation in the face of rapid environmental change.

## Introduction

Gradual and sudden changes in temperature due to anthropogenic activities pose a challenge to organisms, having important consequences on phenotype and fitness (Parmesan 2006; Walker et al. 2019). Ectotherms are especially vulnerable to altered thermal conditions, and exposure to increased temperatures during early development has been shown to lead to phenotypic plasticity in morphology, physiology and behavior, which can impact fitness in a range of taxonomic groups (Valenzuela and Lance 2004; Dang et al. 2015; Dayananda and Webb 2017; Noble et al. 2018; Ślipiński et al. 2021; Raynal et al. 2022). Although research has focused mainly on the effects of early life temperature on individual development, little work has addressed the multifaceted role that environments play across generations (Noble et al. 2018; Du et al. 2023). Environmental stress (e.g. changes in thermal or resource conditions) experienced by mothers is expected to cascade to affect offspring through maternal effects, and this may interact in complex ways with the environments experienced by offspring.

Maternal effects are important contributors to offspring phenotypic variation (Bernardo 1996; Wolf and Wade 2009; Noble et al. 2014), and may moderate the effects of the environment experienced during development. Mothers can adjust their reproductive behavior by altering when and where to nest, by modifying their own body temperature (e.g. Shine and Downes 1999; Doody et al. 2006; Schwanz and Janzen 2008; Refsnider and Janzen 2012; Lorioux et al. 2013; Schwanz 2016; Marco et al. 2018) or differentially investing energy and resources into eggs in response to changes in the environmental conditions they experience (Rutstein et al. 2005; Huang et al. 2013; Carter et al. 2018). For example, nutrient-deprived mothers alter their reproductive allocation compared with mothers fed with normal quality food, leading to changes in offspring phenotype and sex (Warner et al. 2007). Similarly, maternal diet can affect hormone deposition in eggs, which is known to influence hatching success, offspring phenotype and fitness (e.g. Rosenfeld and Roberts 2004; Rutstein et al. 2005; Huang et al. 2013; Warner and Lovern 2014; Carter et al. 2018). Allocation decisions are also expected to be context- and environmentally dependent. For example, females in poor body condition and with limited access to resources are expected to reduce energy investment into reproduction whereas greater investment is expected when resources are plentiful (Griesser et al. 2017; de Zwaan et al. 2019). Maternal effects can therefore exacerbate, dampen, or counteract negative phenotypic or fitness effects of early-life environments experienced by offspring, and may play an important role in explaining the diversity of phenotypic responses observed within and across species (Andrews 2018; Noble et al. 2018; Leivesley and Rollinson 2021; Johnson et al. 2023). This might be especially important in the face of climate change. For instance, a rise in temperature has been predicted to affect feeding behavior and energy intake in ectotherms (Huey and Kingsolver 2019). Food deprivation and diet are linked to investment in eggs and are important factors shaping developmental plasticity in offspring (Van Dyke and Griffith 2018). Therefore, although mothers may behaviorally compensate and reduce incubation temperatures—by nesting in cooler places—this compensation might be limited by several factors, such as the availability of adequate habitat or female body condition. Hence, climate change is likely to result in the co-occurence of environmental conditions that both decrease maternal investment and increase incubation temperatures, highlighting the importance of exploring the interaction between maternal and offspring environments on key fitness traits.

Maternal and offspring environments that impact behavior are likely to have important consequences in shaping how organisms interact with their environment. Such behavioral changes can have a direct link to fitness (reviewed by Saaristo et al. 2018 in association with chemical contamination). Antipredator responses, including predator escape and refuge-seeking, are a case in point because the inability to escape from predatory attacks is inevitably associated with mortality. Thermal developmental conditions and maternal effects are known to affect antipredator strategies. For instance, exposure to high temperatures during early development (e.g. eggs or juveniles) has been shown to affect maximal sprint speed and a range of antipredator behaviors such as predator avoidance and hiding time (Brodie and Russell 1999; Webb et al. 2001; Dalesman and Rundle 2010; McDonald and Schwanz 2018). Similarly, maternal effects can pre-adapt offspring responses to the prevalent predatory conditions through changes in egg composition (Sharda et al. 2021). Such effects are likely mediated by changes in morphology (e.g. size, body condition, etc.), which in turn can influence individual antipredator strategies (Räsänen et al. 2005; Lancaster et al. 2010; Mcghee et al. 2012). Clearly, thermal conditions and maternal effects independently have the potential to influence a range of fitness-related behaviors, however, whether the 2 interact to shape offspring responses remains unknown.

Here, we test whether different developmental temperatures and levels of maternal investment impact morphological and behavioral traits related to antipredator responses in two closely related lizard species, the delicate skink *Lampropholis delicata* and the garden skink, *L. guichenoti*. Both species differ in their life-history strategies with *L. delicata* producing larger clutches of smaller eggs and *L. guichenoti* producing smaller clutches of larger eggs (Forsman and Shine 1995; Qualls and Shine 1997). We apply “phenotypic engineering” methods (Sinervo and Basolo 1996) to manipulate both maternal investment in eggs and offspring temperature in a fully factorial design. Using repeated measures on the same animals we model behavior and performance using Bayesian multivariate mixed models allowing us to partition between and within-individual variance. We predicted that: (1) embryos experiencing high temperatures early in development would be smaller in size and have reduced performance (e.g. Tiatragul et al. 2017; Sanger et al. 2018). As a result, we expect them to be risk adverse relative to embryos experiencing cold temperatures; (2) lower maternal investment in eggs should exacerbate the effects of temperature because less resources would be available for development; and (3) *L. delicata* will be more strongly impacted by temperature and reduced maternal investment because of the smaller egg size (and reduced resources) compared with *L. guichenoti* (Forsman and Shine 1995; Qualls and Shine 1997).

## Methods

### Study species and housing

We used two sympatric skink species. The delicate skink (*Lampropholis delicata*) is a small lizard (max. SVL 51 mm) native to south-eastern Australia (Wilson and Swan 2021). Females lay a single clutch of 3 to 6 small eggs each year. It has been used extensively in experiments to explore how different environments affect the morphology and performance of individuals (Bilcke et al. 2006; Downes and Hoefer 2007; De Jong et al. 2023). In addition, it is an invasive species on some islands (Chapple et al. 2014; Chapple 2016), which suggests that the species has the potential to respond and become used to novel environmental conditions. The garden skink (*Lampropholis guichenoti*) is a small lizard (max. SVL 48 mm) and widespread across south-eastern Australia (Wilson and Swan 2021). Females lay 1 or 2 clutches of 2 to 4 large eggs a year. The garden skink has been used in previous studies exploring the effect of rearing temperatures and humidity on offspring phenotypic plasticity (Qualls and Shine 1998; Booth et al. 2000).

We harmlessly captured 80 gravid female *L. delicata* and *L. guichenoti* using meal worming “fishing” methods in 2 nearby semiurban parks in Sydney (Australia) in December 2020 (coordinates: site 1: −33.91000, 151.18296; and site 2: −33.916928, 151.231015). We captured all the females within 5 days to avoid any time/season-dependent effect in our results. Animals were brought to the laboratory at the Australian National University where they were housed in single-species groups of 5 in indoor terraria (3 to 4 females—width × length: 40 × 55 cm) to allow them to lay eggs. Terraria were filled with approximately 8 cm deep of soil, refuge, a water container, and a container full of vermiculite for egg-laying. Terraria were heated by a lamp and had a UV lamp for UVA/UVB exposure. The heat lamp (25 Watts) was situated at one end to ensure a temperature gradient. Temperatures under the heat lamp range between 28 and 32°C. Lights were set to a photoperiod of 12:12 h (light/dark). Animals were provided with water every day (both spraying the soil and filling the water container). Crickets dusted in calcium and multivitamins were provided every second day. Females were kept in the laboratory for around 2 weeks for egg-laying and then released at their capture locations.

### Experimental design

To explore how incubation temperature and maternal investment interact to affect the performance and antipredator behavior of juveniles, we designed a 2 × 2 fully-factorial experiment where eggs of the 2 skink species were exposed to 2 levels of temperature [cold (23 ± 3 °C) or hot (28 ± 3 °C)] and yolk removal (yolk content reduced or a sham-control) to simulate changes in maternal investment in eggs. Although it would have been interesting to add a “yolk addition” treatment this was not possible because of the risks of fungal and bacterial contamination when using the same syringe twice. Incubation temperatures were selected to mimic extreme temperatures measured in natural nests of *L. delicata* (Cheetham et al. 2011).

Enclosures were checked daily for eggs. We randomly allocated one egg from a clutch to each of our 4 treatments: 23 °C sham-control eggs; 23 °C yolk reduced eggs; 28 °C sham-control eggs; and 28 °C yolk reduced eggs. Our design was a partial split-clutch design as it was not possible to allocate eggs from a given clutch to each of the 4 treatments (i.e. a split-clutch design) given the small clutch sizes of some individuals. For this experiment, we used 22 eggs per treatment combination and species, hatched from 56 and 53 different clutches for each *L. delicata* and *L. guichenoti*, respectively. Eggs allocated to the yolk-reduced treatment were weighed (to the nearest mg) and then pierced with a sterilized insulin syringe to extract part of the yolk. Eggs were weighed again, and the difference in weight pre and postextraction was used as an approximation of the percentage of yolk extracted. Following methods in Sinervo 1990 we aimed for around 15% yolk removal (final result, extracted mean ± SD = 12.49% ± 2.64). Pilot work showed that greater than 15% yolk removal was more likely to result in embryo dislodgement and egg death. Control eggs were weighed and pierced with a needle, but we did not remove any yolk. Eggs were then placed in a container filled with 4 g of vermiculite dampened with 12 g of water and covered with cling-wrap (Glad Wrap) to avoid dehydration. Each egg was then placed in an incubator at the corresponding temperature.

### Measures of morphological traits, performance, and antipredator behavior

We checked the eggs every day for hatchlings and each hatched lizard was individually housed in terraria (20 × 35 cm) heated by a heat cord. Animals also had UV lighting. Enclosures contained paper as a substrate, a water container, and a refuge. All the animals were housed in the same laboratory conditions as adults. Juveniles were fed every second day with pinhead crickets.

Juveniles were measured and their behavior was tested when they were 6 to 12 weeks old (mean age in days ± SD; *L. delicata* cold = 50.66 ± 8.47, hot = 61.20 ± 12.46; *L. guichenoti*: cold = 50.25 ± 7.47, hot = 62.86 ± 12.31). We ensured that there was equal representation from each of the 4 treatment combinations and species (e.g. eggs in cold treatment had longer incubation times) during each measurement session (groups of 48 per day, see below), so we randomly selected individuals from the pool of available lizards (we had more animals available than needed for this study because of other experiments). Body size and mass can influence performance and behavior (Huey and Hertz 1984; Baxter-Gilbert et al. 2018). As such, we measured weight, snout-vent length, tail length, and total length at each measurement to control for these during the analysis. Given the small size of hatchlings (mean SVL ± SD = 18.47 ± 1.203 mm), we weighed lizards (to the nearest mg) using an Ohaus scale and took a ventral photo of each lizard against grid paper (1 mm squares). From this photo, we later measured the snout-vent length (SVL) as the distance (in mm) from the snout to the cloaca, and the tail length as the distance from the cloaca to the tip of the tail using imageJ (Abràmoff et al. 2004).

We set up 12 CCTV cameras with each recording 4 individual terraria simultaneously. Each week we selected 48 juveniles from the correct age window—a mix of the 2 species and 4 treatment combinations. We weighted and photographed the animals, and then placed them randomly across the shelves to avoid any biases associated with the location in the laboratory. The terraria for the assays were opaque to avoid lizards viewing each other which could influence their behavior. In addition, the terraria were separated from the walking corridors by thick-opaque curtains to avoid the presence of the researchers influencing lizard behavior. All enclosures had a refuge and a water container. A heat lamp was placed on one side of the enclosure to ensure a thermal gradient of at least 6° between the lamp and the refuge. The same group of animals were housed in these terraria during the 6 days of trials.

Over the 6 days that animals remained under cameras we took 3 measurements of performance and antipredator behavior for each animal on alternate days (i.e. day 1 performance, day 2 antipredator behavior, 3 times). We followed the same order for all batches of lizards. More specifically, we collected the following behavioral variables:

[1] *Running speed (in seconds)*: We measured running performance as the total time needed to run the 1-m-long straight racetrack. We also recorded burst speed as the fastest 25 cm section (the racetrack had a detector and time tracker every 25 cm). For analysis, we used the total time used to cover the full one meter and the fastest time taken to run a 25 cm interval. Fifteen minutes before each performance measure, lizards were placed in an incubator at 28 °C to ensure constant body temperature across lizards. Although recent studies have shown that the thermal optimum is slightly higher than 28 °C (Anderson et al. 2023; Zhang et al. 2023) there is substantial individual and species-specific variation with 28 °C being firmly within this range. Animals were then transferred to the racetrack and were chased down toward a terrarium placed at the end of the track. After the trial, each animal was placed back in their corresponding terraria. We repeated the running trial 3 times for each individual, on alternate days.
[2] *Activity*: We measured the distance traveled (in cm) as a proxy for the activity level of individuals. To record the activity, we removed the refuge and the water container from the terraria to avoid animals hiding. We then switched on the camera and left the animals to behave and move freely in their terrariums for 20 min. The distance covered was later calculated using the software EthoVision XT (vers. 12.0).
[3] *Antipredator behavior*. Immediately after the activity trial, we replaced the refuge. After 30 min, we simulated a predatory attack. To do this, we approached the terraria and tapped the animals with a paint brush near the tail until they took refuge. The same person (JZ) performed all the predatory attacks. The only identification in the terraria was the randomly allocated ID number provided to the eggs at the beginning of the experiment such that JZ was blind to the treatment. After the simulated attack, we recorded each lizard’s response for 90 min. From the videos we calculated (1) the time (in s) each lizard took to seek refuge because the first tap in the tail (hereafter “time to hide”) (2) the time between the moment the animal took refuge (time to hide) until the animal’s head appeared at the entrance and was clearly visible in the videos (hereafter “hiding time,” in seconds) and (3) the time elapsed between when the animal took refuge (time to hide) to the moment the animal left the refuge to start their normal activity after the predatory attack (hereafter “time to activity”). We consider this to be the moment when the back forelimbs left the refuge. We repeated the activity and antipredator assays three times on alternate days from performance trails.

All trials took place in January and February 2021, between 9 and 12 am, during the period when activity was the highest. We recorded 22 individuals per species and treatment. We discarded from the analysis any individual that lost their tail during the experiment (*n* = 5 out of 176) to avoid any bias associated with potentially impaired running ability (Cromie and Chapple 2012). The final sample sizes were 22 for hot-control *L. delicata*, cold-control and hot-yolk-reduced *L. guichenoti*, and 21 for the rest of the combinations.

### Statistical analysis

We used Bayesian Multivariate Mixed Effects Models using *rstan* (Stan Development Team 2020) in the package *brms* (Bürkner 2018) to explore whether incubation temperature and maternal investment impacted morphology (tail length, SVL, and weight), performance (running speed and activity level) and antipredator behavior. We also estimated the correlations between the variables measured at the between- and within-individual levels. We first checked for the normality of the data by visualizing the residuals of intercept-only random effects model. All models included 2 crossed-random effects: a random intercept for each lizard and a random intercept to control for between clutch effects. To meet the assumptions of normality running speed (both 25 cm burst and 1 m long) was log-transformed for the 2 species. For all models, we ran 4 MCMC chains, with each chain being run for 4000 iterations with a warmup of 1000 using default priors. RStan implements a Hamiltonian Markov process for each MCMC chain. This has been shown to perform well allowing one to fit Bayesian models with fewer MCMC chains and less burnin/warmup because it minimizes inefficiencies in MCMC random walks (Bürkner 2017; Monnahan et al. 2017). We retained each sample (thinning of 1) from each chain. We checked that MCMC chains were mixing well by visualizing trace plots, checked that all chains had converged (*R*_hat_ < 1.01) and that the effective sample size for each parameter were greater than 1000.

Both species were analyzed separately. We ran 2 separate multivariate mixed models for each species, 1 with morphological traits, and the other with all performance and antipredator behavior as response variables. We separated morphology from performance and behavior because the latter variables were measured 3 times allowing us to decompose between and within-individual variation (O’Dea et al., 2022). Missing data resulted from video failures or lack of performance in some assays (see Supplementary Table S1 for percent missing data). Instead of a complete case analysis, we retained missing data and used data augmentation methods during model fitting which can be more powerful than complete case analyses (Noble and Nakagawa, 2021). Models contained fixed effects (explanatory variables) of incubation temperature and maternal investment treatment along with their interaction. We also included individual and clutch identities as random effects (intercepts). In the morphology model, only clutch was added as random effect given that we only had a single measurement for each individual. In the behavior model, SVL was included as covariate to control for any potential effect of body size on the traits measured. We repeated the behavior/performance model without SVL as a covariate to explore for any indirect effect of temperature and maternal treatments on behavior that might have been influenced by body size. Using the posterior distributions from these models, we derived the key interaction comparison of interest—whether the difference between control and yolk removal treatments was amplified or subdued in response to temperature. In addition, we used the posterior distribution to calculate the overall temperature and maternal investment effect by pooling the posteriors across the second factor.

Repeatability of behavioral traits was calculated from intercept-only models using the posterior distribution for each variance estimate as follows: $\sigma_{id}^{2}$ / $\sigma_{id}^{2}$ + $\sigma_{clutch}^{2}$ + $\sigma_{w}^{2}$, where $\sigma_{id}^{2}$ is the between-individual variance, $\sigma_{clutch}^{2}$ is the between clutch variance, and $\sigma_{w}^{2}$ is the residual variance. We present the posterior mean and 95% credible intervals (CI) for parameters of interest. Credible intervals not overlapping zero were considered significant and we calculate and present the probability (p_MCMC_) of obtaining this effect under a null hypothesis of no effect.

## Results

Most of the performance and antipredator variables showed moderate to high repeatability between the three different measures taken (Supplementary Table S2).

### Maternal investment and early thermal environment affected morphology in *L guichenoti* but not *L. delicata*

**Fig. 1.**
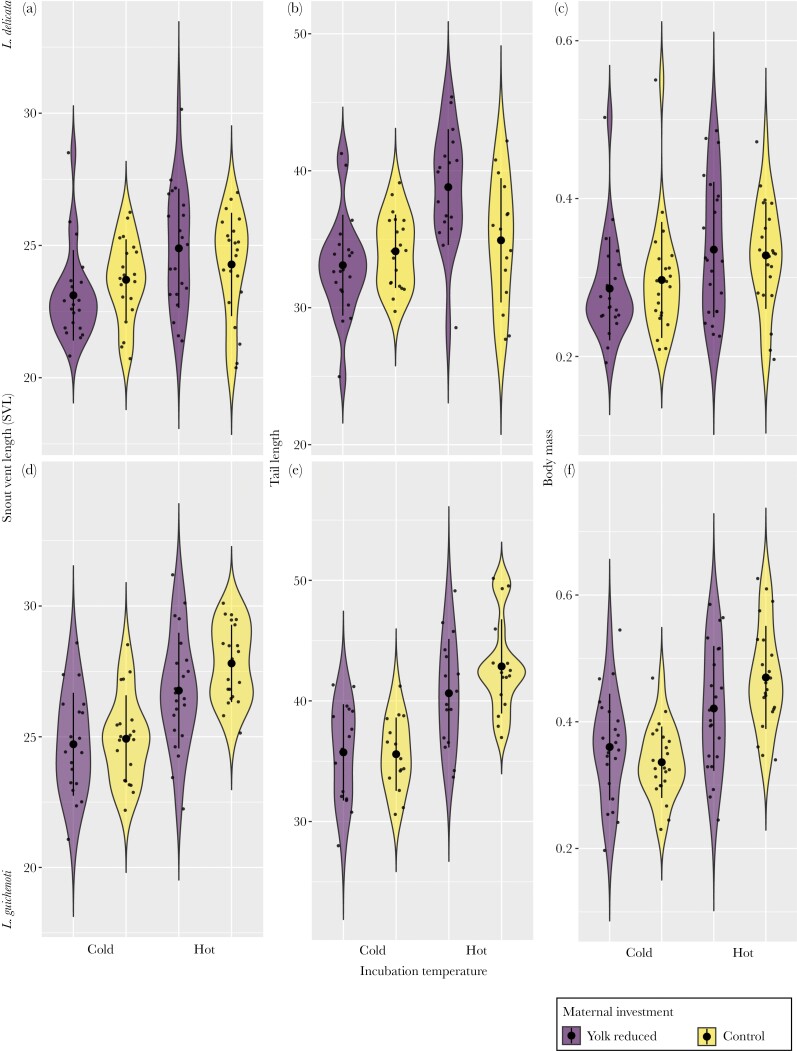
Effect of temperature (cold, 23 °C vs hot, 28 °C) and maternal resource investment (yolk reduced vs control) on morphological traits of *L. delicata* (a, b and c) and *L. guichenoti* (d, e and f). See Table 1 for relevant statistics.

We did not find any effect of temperature, maternal investment, or their interaction on morphology in *Lampropholis delicata* [tail length: (Fig 1a), snout-vent-length (SVL, Fig. 1b) or weight (Fig. 1c) (see Table 1)].

**Table 1:** Posterior means and 95% credible intervals for the interaction between temperature (Temp) and maternal investment (Invest) along with the main effects of temperature and maternal investment on morphological traits for *Lampropholis delicata* and *Lampropholis guichenoti*. Main effects are pooled posterior means over each level of the second predictor variable (either temperature or maternal investment treatments depending on the focal variable). Posterior distributions are estimated from a multi-response model, which accounts for the correlation between morphological traits. Bold indicates significant effects.

Species	Trait	Term	Estimate	2.5%	97.5%	pMCMC
*L. delicata*	SVL	Interaction [(C23 to A23) to (C28 to A28)]	−0.198	−1.651	1.288	0.790
		Temp (23 to 28)	−0.192	−1.327	0.913	0.738
		Invest (C to A)	0.404	−0.663	1.442	0.438
	Weight	Interaction [(C23 to A23) to (C28 to A28)]	0.010	−0.035	0.057	0.665
		Temp (23 to 28)	−0.006	−0.042	0.031	0.728
		Invest (C to A)	0.002	−0.031	0.038	0.918
	Tail	Interaction [(C23 to A23) to (C28 to A28)]	−2.898	−6.238	0.516	0.089
		Temp (23 to 28)	−1.299	−4.979	2.128	0.565
		Invest (C to A)	−0.777	−4.348	2.475	0.768
*L. guichenoti*	SVL	Interaction [(C23 to A23) to (C28 to A28)]	1.324	−0.501	3.124	0.150
		**Temp (23 to 28)**	−**2.973**	−**4.715**	−**1.241**	**0.000**
		Invest (C to A)	0.348	−1.394	2.072	0.739
	Weight	**Interaction [(C23 to A23) to (C28 to A28)]**	**0.089**	**0.006**	**0.172**	**0.035**
		**Temp (23 to 28)**	−**0.112**	−**0.207**	−**0.019**	**0.012**
		Invest (C to A)	0.014	−0.080	0.108	0.862
	Tail	Interaction [(C23 to A23) to- (C28 to A28)]	2.174	−1.420	5.776	0.241
		**Temp (23 to 28)**	−**6.347**	−**9.619**	−**3.079**	**0.000**
		Invest (C to A)	0.902	−2.361	4.147	0.627

In contrast, maternal investment and temperature treatment interacted to affect weight in *L guichenoti* (Table 1). The weight difference between control and yolk removal treatment was larger in the cold incubation temperature compared with the difference under warm incubation temperatures for *L guichenoti*. We also found a statistically significant effect of incubation temperature on juvenile size of *L. guichenoti.* Generally, individuals coming from eggs incubated at hotter temperatures had longer tails (estimate = 6.347, 95% CI = 3.079 to 9.619, Fig. 1d), larger SVL (estimate = 2.973, 95% CI = 1.241 to 4.715, Fig. 1e) and were heavier (estimate = 0.112, 95% CI = 0.019 to 0.207, Fig. 1f) than those hatched from eggs at colder temperatures (Table 1).

In both species, there was a positive correlation among SVL, tail length, and body mass, with individuals with larger bodies also being heavier and having longer tails (Supplementary Table S3).

### Impacts of maternal investment on running performance are mediated by early thermal environment in both species

**Fig. 2.**
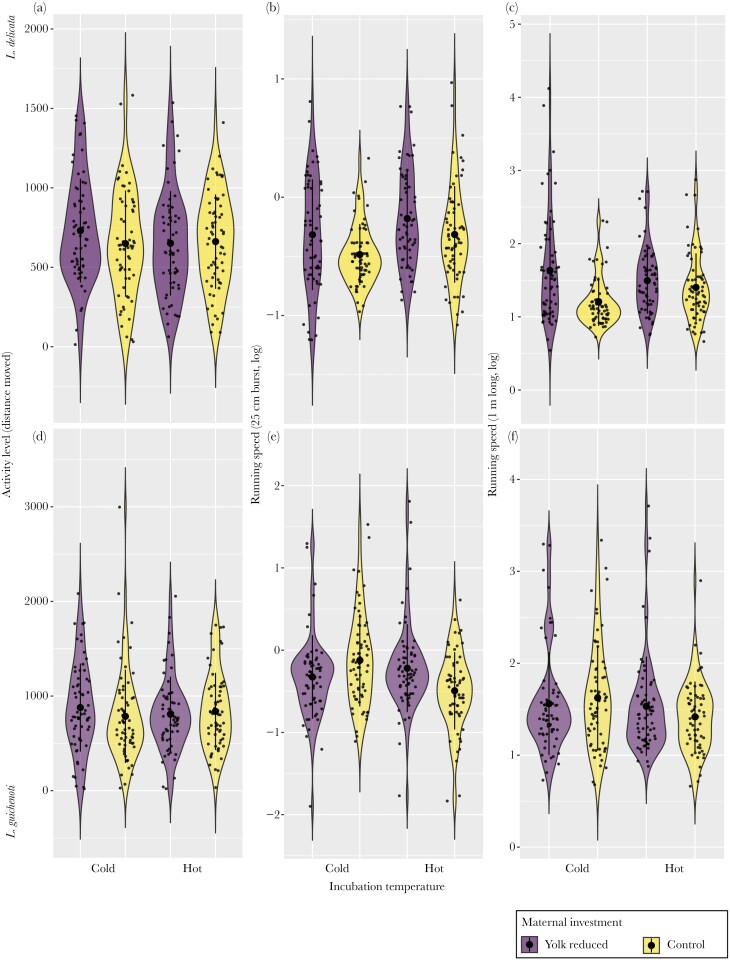
Effect of temperature (cold, 23 °C vs hot, 28 °C) and maternal resource investment (yolk reduced vs control) in lizard performance: distance moved and running speed (25 cm burnst and 1 m). *L. delicata* panels a, b and c, and *L. guichenoti* panels d, e and f. Note: 3 datapoints (raw data > 10 s and < 0.01 s) in the 25 burst of *L. delicata* were removed for visualization reasons.

We found a statistically significant interaction between maternal yolk investment and incubation temperature for the 25 cm burst speed of juvenile *L. guichenoti* (Fig. 2d, Table 1). This effect persisted even when controlling for SVL (Supplementary Table S4). The difference in 25 cm burst speed between control and yolk removal eggs was significantly larger under hot incubation conditions compared with cold conditions (Table 2—estimate = −0.470, 95% CI: −0.854 to −0.081, pMCMC = 0.018). In cold incubation treatments, lizards from the control tended to have a higher burst speed compared with those from yolk-reduced treatment, whereas the opposite was true for hot incubated lizards (Fig. 2).

**Table 2:** Posterior means and 95% credible intervals for the interaction between temperature (Temp) and maternal investment (Invest) along with the main effects of temperature and maternal investment on behavioral and performance traits for *Lampropholis delicata* and *Lamprpholis guichenoti*. The main effects are pooled posterior means over each level of second predictor variable. Estimates are from a Bayesian multivariate (multi-response) model not controlling for SVL. See Supplement for the model with SVL controlled. Bold estimates are significant and italics indicated effects with less than a 10% chance of being observed.

Species	Trait	Term	Estimate	Q2.5	Q97.5	pMCMC
*L. delicata*	Time to activity (s)	*Interaction [(C23 to A23) to (C28 to A28)]*	−*864.379*	−*1847.746*	*103.120*	*0.080*
		Temp (23 to 28)	−676.650	−1696.061	325.761	0.239
		Invest (C to A)	233.230	−826.597	1216.182	0.731
	Hiding time (s)	Interaction [(C23 to A23) to (C28 to A28)]	−718.025	−1631.020	192.257	0.117
		Temp (23 to 28)	−502.792	−1406.149	388.546	0.328
		Invest (C to A)	115.504	−810.196	985.614	0.830
	Distance m (cm)	Interaction [(C23 to A23) to (C28 to A28)]	35.435	−174.231	252.904	0.748
		Temp (23 to 28)	33.968	−121.384	187.916	0.660
		Invest (C to A)	−50.048	−204.844	109.218	0.528
	log 1m Speed (cm/s)	*Interaction [(C23 to A23) to (C28 to A28)]*	*0.335*	*-0.023*	*0.689*	*0.068*
		Temp (23 to 28)	−0.028	−0.405	0.349	0.928
		Invest (C to A)	−0.262	−0.644	0.116	0.233
	Log burst speed (cm/s)	Interaction [(C23 to A23) to (C28 to A28)]	0.214	−0.178	0.591	0.279
		Temp (23 to 28)	−0.062	−0.398	0.270	0.737
		Invest (C to A)	−0.095	−0.435	0.238	0.610
*L. guichenoti*	Time to activity (s)	Interaction [(C23 to A23) to (C28 to A28)]	−93.237	−1263.710	1052.332	0.876
		Temp (23 to 28)	−42.896	−870.269	778.343	0.929
		Invest (C to A)	133.420	−681.068	951.159	0.750
	Hiding time (s)	Interaction [(C23 to A23) to (C28 to A28)]	166.056	−694.735	989.883	0.683
		Temp (23 to 28)	−2.416	−631.727	619.633	0.992
		Invest (C to A)	90.790	−519.904	697.379	0.767
	Distance moved (cm)	Interaction [(C23 to A23) to (C28 to A28)]	127.921	−185.868	444.379	0.423
		Temp (23 to 28)	4.077	−249.201	263.186	0.976
		Invest (C to A)	−25.340	−282.031	232.134	0.843
	Log 1 m speed (cm/s)	Interaction [(C23 to A23) to (C28 to A28)]	−0.179	−0.549	0.206	0.348
		Temp (23 to 28)	0.116	−0.198	0.432	0.489
		Invest (C to A)	−0.027	−0.347	0.281	0.878
	Log burst speed (cm/s)	**Interaction [(C23 to A23) to (C28 to A28)]**	−**0.470**	−**0.854**	−**0.081**	**0.018**
		Temp (23 to 28)	0.134	−0.327	0.603	0.774
		Invest (C to A)	−0.034	−0.502	0.424	0.953

Although not significant, we also found a similar interaction between maternal yolk investment and incubation temperature on 1m sprint speed in *L. delicata* (Table 2 and Supplementary Table S4). However, in contrast to *L. guichenoti*, there was a significantly smaller difference in 1 m burst speed between control and yolk removal eggs in lizards incubated under hot conditions compared with cold conditions (Table 2—estimate = 0.335, 95% CI: −0.023 to 0.689, pMCMC = 0.068).

In both species, trials where individuals had a faster burst speed were also quicker to travel the full 1m track (*L. delicata*: within-individual correlation ± SE = 0.42, 95% CI = 0.30 to 0.54; *L. guichenoti*: within-individual correlation ± SE = 0.51, 95% CI = 0.4 to 0.61). In addition, individuals that had faster burst speed also tended to run the full 1 m faster (*L. delicata*: between-individual correlation = 0.82, 95% CI = 0.63 to 0.95; *L. guichenoti*: between-individual correlation = 0.95, 95% CI = 0.89–0.99).

### Weak evidence that antipredator behavior is affected by early thermal environment and maternal investment

Antipredator behaviors were weakly integrated with performance measures at the between and within-individual levels for most traits (Supplementary Table S5). At the between-individual level, there was a strong correlation (*r* = 0.90, 95% CI: 0.78 to 0.97) between the hiding time and the time to activity as well as between the time to activity and burst speed (*r* = 0.32, 95% CI: 0.04 to 0.57) in *L. guichenoti*. At the within-individual level trials lizards with shorter hiding times also resumed their activity faster overall for both species (*L. guichenoti*: *r* = 0.69, 95% CI: 0.58 to 0.77; *L. delicata*: *r* = 0.82, 95% CI: 0.73 to 0.88).

**Fig. 3.**
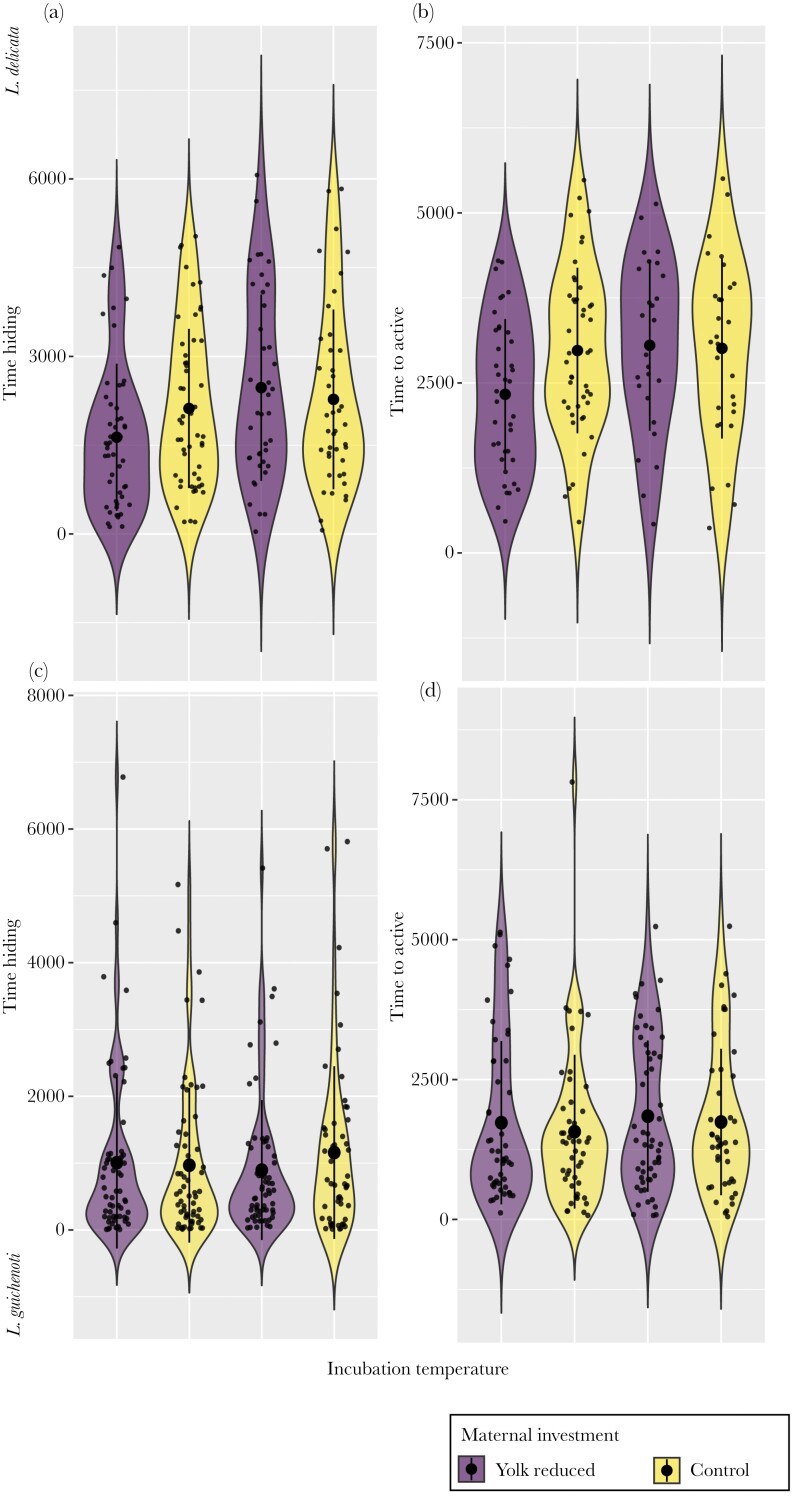
Effect of temperature (cold, 23 °C vs hot, 28 °C) and maternal resource investment (yolk reduced, control) in lizard antipredator behavior: time hiding (time, in seconds, since the lizard hided in the refuge until the head was visible) and time to active (time before resuming activity). *L. delicata* panels a and b, and *L. guichenoti* panels c and d.

Changes in the time to activity after a simulated predatory attack between control and yolk removal eggs depended on temperature in *L. delicata* when controlling for body size (interaction estimate = −1003.752, 95% CI = −1988.452 to −33.590, pMCMC = 0.044, Supplementary Table S4). Similar effects were observed when not controlling for body size, but it was not significant (Table 2). Yolk-reduced lizards appeared to resume activity faster compared with lizards hatching from control eggs when incubated at cold temperatures whereas there was no difference between control and yolk-removed eggs under hot temperatures (Fig. 3b). We did not find strong evidence that other behavioral traits involved in antipredator responses were impacted by temperature, maternal investment, or their interaction in *L. delicata* or *L. guichenoti* (Table 2 and Supplementary Table S4; Fig. 3).

## Discussion

Environmental conditions experienced by offspring are expected to interact in complex ways with the environments experienced by their parents. Anthropogenic climate change and other human-associated stressors will simultaneously alter temperatures impacting other factors such as maternal food availability and stress levels that could affect the maternal investment in eggs. In ectotherms, temperature conditions during early life are known to have important effects on individuals (Noble et al. 2018; While et al. 2018; Singh et al. 2020). but little work has explored the interaction between maternal investment and temperatures; even though there have been suggestions that these factors explain variations in thermal effects (Noble et al. 2018). Understanding how maternal effects interact with offspring environments to affect development and fitness is important for ascertaining whether climate-induced changes are likely to be exacerbated or dampened by impacts in parental generations and helps to explain why responses vary so dramatically across populations and species with respect to early thermal conditions. Here we explored whether cold and hot incubation temperatures lead to different responses in morphology, performance, and antipredator behavior in 2 sympatric skink species (*L. delicata* and *L. guichenoti*), and whether a controlled manipulation in the amount of yolk in eggs moderated such responses. We found that incubation temperature affects morphology, and that temperature interacts with maternal yolk treatment to moderate performance and the associated antipredator responses. The effects observed appear to be species-specific, and possibly the result of different life-history strategies.

### Effect of early life thermal conditions and maternal investment on morphology

Contrary to our expectations we found that juveniles of *L. guichenoti* emerging from eggs incubated at hot temperatures were larger and heavier than those reared at cold temperatures, however, incubation temperature did not significantly affect morphology in *L. delicata* (see also de Jong et al. 2022). Because we did not find any effect of incubation temperature on body size on the day of hatch in either of the 2 species (data collected for another study), our results show that the difference in body size between individuals coming from cold and hot incubation regimes in *L. guichenoti* appeared during early juvenile development. Our findings suggest that, rather than a change in metabolic and developmental rates during embryo growth of this species, incubation temperature programmed posthatching metabolism, potentially affecting feeding and growth rates, as observed in previous studies in lizards exposed to high temperatures (Singh et al. 2020).

We did not find any effect of the maternal investment treatment on body length for either *L. guichenoti* or *L. delicata*, but body mass was affected in *L. guichenoti*. Although we found that individuals of both species hatched smaller from the yolk-reduced treatment than from the control (again, data collected for another study, but see also e.g. Warner and Lovern 2014), maternal investment effects on morphology seem to have disappeared by the age tested. This finding contrasts with previous studies where juveniles from yolk-reduced treatments hatched smaller but also showed slower growth rates than those from control treatments (Warner and Lovern 2014). Our results are not completely surprising, however, because many of the impacts of incubation conditions on morphological and behavioral traits observed in recently hatched individuals often disappear as the individual's age (Pearson and Warner 2016; McDonald and Schwanz 2018). This could also be the case for many maternal effects. In our study, the fact that juveniles of both yolk-reduced and control eggs attain a similar body size a few weeks after hatching suggests that individuals can, to some extent, compensate for the poor start in life by accelerating their growth during the first few weeks, probably by increasing their feeding rates. This might be key, because in a range of species, larger juvenile body size has been associated with better survival (e.g. Einum and Fleming 2000; Webb et al. 2006), although this relationship is sometimes complex and dependent of other ecological and biological factors (Sinervo et al. 1992; Warner and Andrews 2002; Langkilde and Shine 2005). However, it is possible that the observed compensation growth was allowed by the ad libitum feeding in the laboratory conditions, so future studies could explore the potential of this compensation to happen in a field setup where the food will be more limiting.

### Effect of early life thermal conditions and maternal investment on performance and behavior

The growth compensation observed in the analyses of morphology might be associated with changes in the allocation of resources and trade-offs with other fitness-associated traits during development, such as the observed slower running speed of juveniles of *L. delicata* hatched from yolk-reduced eggs and of *L. guichenoti* hatched from yolk-reduced eggs in cold incubation conditions. This result suggests that, even though animals from poor developmental conditions are able to morphologically catch up with individuals from more beneficial early-life conditions, this might come at a cost in performance. This is in accordance with previous studies on *Sceloporus undulatus* showing that clutches with individuals with fast growth rates, but slow runners, had lower survival than slow-growing but fast runners (Warner and Andrews 2002).

Impaired performance was expected to lead to more risk-adverse individuals to compensate for a potential increased vulnerability to predators. In contrast to our prediction, antipredator behavior of *L. guichenoti* was not affected by our treatments. In addition, we found that juvenile *L. delicata* from the yolk-reduced treatments, especially when reared at cold temperatures, took shorter to resume activity after a simulated predatory attack than those from the control treatment—although this was not a strong effect and only significant when body size was added as a covariate in the models. After accounting for their body size, our results show that despite their impaired performance, juveniles from the yolk-reduced treatment leave the refuge quicker than those from the control treatment, suggesting that the treatments shape antipredator behaviors in a way that is independent of the individuals’ size. Our results contrast with previous studies that have found that individuals with perceived higher vulnerability alter their antipredator behavior to hide for longer to successfully avoid a predatory attack (e.g. Martin and López 1999; Cooper 2007; Iglesias-Carrasco et al. 2016). Instead, our results suggest that the benefits of resuming activities, such as basking and feeding, might potentially outweigh the potential survival costs in *L. delicata*, at least in a laboratory setting where real predators are absent. However, from our experiment, we cannot know whether the change in antipredator response observed confers a fitness advantage regarding, for example, quicker growth, or instead would lead to costs in terms of increased predation risk in the wild. Further experiments would benefit from studying how incubation temperature and maternal investment interact to affect behavior, and the consequent fitness payoffs, in a more natural setting.

### Species-specific responses

The 2 skink species studied differed in their morphological, performance, and antipredator responses to the incubation temperature and maternal investment. These differences could be in part associated with some life-history traits, such as the size of the egg. Eggs of *L. delicata* are smaller than those of *L. guichenoti*, which might make these eggs more sensitive to small alterations in the incubation environment, strongly impacting the phenotype and behavior of juveniles, as observed in our study (see also Thompson et al. 2001). Although this result suggests that environmental changes in the early thermal environment coupled with the reduced maternal investment will impact *L. delicata* more negatively compared with *L. guichenoti,* we caution over interpretation because phenotypic and behavioral plasticity could provide juveniles with increased environmental tolerance that may confer a fitness benefit in face of climate change (DeWitt et al. 1998; Fox et al. 2019; Yeh and Price 2004; but see Oostra et al. 2018). The ability to plastically respond to different environmental conditions could also explain *L. delicata*’s success as an invasive species (see e.g. Davidson et al., 2011). In contrast, the lack of behavioral responses in *L. guichenoti* could be a sign of the inability of the species to adaptively react to environmental challenges, or rather suggest that in this species the explored behavioral responses might not impose a fitness cost. Because the incubation temperatures used in our experiment overlap with those occurring in the wild (Cheetham et al. 2011), it will be interesting to explore how more extreme thermal incubation conditions, expected as a consequence of anthropogenic climate change, will interact with the maternal condition to shape hatchling performance and survival in the future.

### Conclusions

We have shown that exploring the complex interaction between offspring and maternal environments can be critical to predict how anthropogenic activities will affect individual performance and ultimately fitness. Although maternal yolk investment did not buffer the effects of incubation temperature in all the morphological and behavioral traits measured, the general pattern suggests that a reduction in the resources allocated by mothers to eggs had a limited effect on offspring traits at the high temperature, but exacerbates the response triggered by colder incubation temperatures. This suggests that these skink species are adapted to develop under slightly warm conditions and that such warm conditions allow them to some extent buffer any potential negative consequences of the reduction in maternal investment. However, the incubation temperatures used in this study are within the range of those experienced in the wild. Future research will benefit from exploring how higher temperatures than those explored here, will affect offspring fitness in the face of the extreme temperatures predicted by climate change. In addition, research will benefit from studying whether any plastic responses are adaptive in novel environment conditions and whether species sensitivity depends on species-specific life history.

## Supplementary Material

arae035_suppl_Supplementary_Material

## Data Availability

Analyses reported in this article can be reproduced using the data provided by Iglesias-Carrasco et al (2024). Code and data used in this article can be accessed via Zenodo (https://zenodo.org/records/11112408) or GitHub, https://github.com/daniel1noble/lampro_yolk_behav.

## References

[CIT0001] Abràmoff MD , Magalhães PJ, Ram SJ. 2004. Image processing with imageJ. Biophotonics International 11(7):36–41. https://iro.uiowa.edu/esploro/outputs/journalArticle/Image-processing-with-ImageJ/9984209058802771

[CIT0002] Anderson RO , Tingley R, Hoskin CJ, White CR, Chapple DG. 2023. Linking physiology and climate to infer species distributions in Australian skinks. J Anim Ecol. 92(10):2094–2108. 10.1111/1365-2656.14000 37661659

[CIT0003] Andrews RM. 2018. Developmental plasticity in reptiles: insights into thermal and maternal effects on chameleon phenotypes. J Exp Zool A Ecol Integr Physiol. 329(6-7):298–307. 10.1002/jez.2160 29682910

[CIT0004] Baxter-Gilbert J , Riley JL, Whiting MJ. 2018. Runners and fighters: clutch effects and body size drive innate antipredator behaviour in hatchling lizards. Behav Ecol Sociobiol. 72(6):97. 10.1007/s00265-018-2505-7

[CIT0005] Bernardo J. 1996. Maternal effects in animal ecology. Am Zool. 36(2):83–105. 10.1093/icb/36.2.83

[CIT0006] Bilcke J , Downes S, Büscher I. 2006. Combined effect of incubation and ambient temperature on the feeding performance of a small ectotherm. Austral Ecol. 31(8):937–947. 10.1111/j.1442-9993.2006.01663.x

[CIT0007] Booth DT , Thompson MB, Herring S. 2000. How incubation temperature influences the physiology and growth of embryonic lizards. J Comp Physiol B Biochem Syst Environ Physiol. 170(4):269–276. 10.1007/s003600000097 10935517

[CIT0008] Brodie ED , Russell NH. 1999. The consistency of individual differences in behaviour: Temperature effects on antipredator behaviour in garter snakes. Anim Behav. 57(2):445–451. 10.1006/anbe.1998.0990 10049485

[CIT0009] Bürkner PC. 2017. brms: an R package for Bayesian multilevel models using Stan. J Stat Softw. 80(1):1–28. 10.18637/jss.v080.i01

[CIT0010] Bürkner PC. 2018. Advanced Bayesian multilevel modelling with the R package brms. R J 10(1):395–411. 10.32614/rj-2018-017

[CIT0011] Carter AW , Bowden RM, Paitz RT. 2018. Evidence of embryonic regulation of maternally derived yolk corticosterone. J Exp Biol. 221(Pt 22):jeb182600. 10.1242/jeb.182600 30266787 PMC6262762

[CIT0012] Chapple DG , Reardon JT, Peace, JE. 2016. Origin, spread and biology of the invasive plague skink (*Lampropholis delicata*) in New Zealand. In: Chapple D, editor. New Zealand Lizards. Springer. p. 341–360. 10.1007/978-3-319-41674-8

[CIT0013] Chapple DG , Miller KA, Chaplin K, Barnett L, Thompson MB, Bray RD. 2014. Biology of the invasive delicate skink (*Lampropholis delicata*) on Lord Howe Island. Aust J Zool. 62(6):498–506. 10.1071/zo14098

[CIT0014] Cheetham E , Doody JS, Stewart B, Harlow P. 2011. Embryonic mortality as a cost of communal nesting in the delicate skink. J Zool. 283(4):234–242. 10.1111/j.1469-7998.2010.00764.x

[CIT0015] Cooper WE. 2007. Compensatory changes in escape and refuge use following autotomy in the lizard *Sceloporus virgatus*. Can J Zool. 85(1):99–107. 10.1139/Z06-200

[CIT0016] Cromie GL , Chapple DG. 2012. Impact of tail loss on the behaviour and locomotor performance of two sympatric *Lampropholis* skink species. PLoS One. 7(4):e34732. 10.1371/journal.pone.0034732 22523555 PMC3327716

[CIT0017] Dalesman S , Rundle SD. 2010. Influence of rearing and experimental temperatures on predator avoidance behaviour in a freshwater pulmonate snail. Freshw Biol. 55(10):2107–2113. 10.1111/j.1365-2427.2010.02470.x

[CIT0018] Dang W , Zhang W, Du WG. 2015. Incubation temperature affects the immune function of hatchling soft-shelled turtles, *Pelodiscus sinensis*. Sci Rep. 5(1):1–9. 10.1038/srep10594 PMC4450580 26028216

[CIT0019] Davidson A , Jennions M, Nicotra A. 2011. Do invasive species show higher phenotypic plasticity than native species and, if so, is it adaptive? A meta-analysis. Ecology Lett. 14:419–431. 10.1111/j.1461-0248.2011.01596.x 21314880

[CIT0020] Dayananda B , Webb JK. 2017. Incubation under climate warming affects learning ability and survival in hatchling lizards. Biol Lett. 13(3):20170002. 10.1098/rsbl.2017.0002 28298595 PMC5377038

[CIT0022] de Jong MJ , White CR, Wong BBM, Chapple DG. 2022. Univariate and multivariate plasticity in response to incubation temperature in an Australian lizard. J Exp Biol. 225(22):jeb244352. 10.1242/jeb.244352 36354342 PMC10112869

[CIT0021] De Jong MJ , Alton LA, White CR, O’Bryan MK, Chapple DG, Wong BBM. 2023. Long-term effects of incubation temperature on growth and thermal physiology in a small ectotherm. Philos Trans R Soc London Ser B. 378(1884):20220137. 10.1098/rstb.2022.0137 37427479 PMC10331899

[CIT0023] de Zwaan DR , Camfield AF, MacDonald EC, Martin K. 2019. Variation in offspring development is driven more by weather and maternal condition than predation risk. Funct Ecol. 33(3):447–456. 10.1111/1365-2435.13273

[CIT0024] DeWitt TJ , Sih A, Wilson DS. 1998. Cost and limits of phenotypic plasticity. Trends Ecol Evol. 13(2):77–81. 10.1016/s0169-5347(97)01274-3 21238209

[CIT0025] Doody JS , Guarino E, Georges A, Corey B, Murray G, Ewert M. 2006. Nest site choice compensates for climate effects on sex ratios in a lizard with environmental sex determination. Evol Ecol. 20(4):307–330. 10.1007/s10682-006-0003-2

[CIT0026] Downes S , Hoefer AM. 2007. An experimental study of the effects of weed invasion on lizard phenotypes. Oecologia 153(3):775–785. 10.1007/s00442-007-0775-2 17541644

[CIT0027] Du WG , Li SR, Sun BJ, Shine R. 2023. Can nesting behaviour allow reptiles to adapt to climate change? Philos Trans R Soc B Biol Sci. 378(1884):15–17. 10.1098/rstb.2022.0153 PMC10331901 37427463

[CIT0028] Einum S , Fleming IA. 2000. Selection against late emergence and small offspring in Atlantic salmon (*Salmo salar*). Evolution. 54(2):628–639. 10.1111/j.0014-3820.2000.tb00064.x 10937238

[CIT0029] Forsman A , Shine R. 1995. Parallel geographic variation in body shape and reproductive life history within the Australian scincid lizard *Lampropholis delicata*. Funct Ecol. 9(6):818–828. 10.2307/2389979

[CIT0030] Fox RJ , Donelson JM, Schunter C, Ravasi T, Gaitán-Espitia JD. 2019. Beyond buying time: the role of plasticity in phenotypic adaptation to rapid environmental change. Philos Trans R Soc B: Biol Sci. 374(1768):20180174. 10.1098/rstb.2018.0174 PMC6365870 30966962

[CIT0031] Griesser M , Wagner GF, Drobniak SM, Ekman J. 2017. Reproductive trade-offs in a long-lived bird species: condition-dependent reproductive allocation maintains female survival and offspring quality. J Evol Biol. 30(4):782–795. 10.1111/jeb.13046 28135017

[CIT0032] Huang V , Bowden RM, Crews D. 2013. Yolk-albumen testosterone in a lizard with temperature-dependent sex determination: Relation with development. Gen Comp Endocrinol. 186:67–71. 10.1016/j.ygcen.2013.02.019 23467072

[CIT0033] Huey RB , Hertz PE. 1984. Effects of body size and slope on acceleration of a lizard (*Stellio stellio*). J Exp Biol. 110(1):113–123. 10.1242/jeb.110.1.113

[CIT0034] Huey RB , Kingsolver JG. 2019. Climate warming, resource availability, and the metabolic meltdown of ectotherms. Am Naturalist. 194(6):E140–E150. 10.1086/705679 31738103

[CIT0035] Iglesias-Carrasco M , Head ML, Cabido C. 2016. Habitat dependent effects of experimental immune challenge on lizard anti-predator responses. Behav Ecol Sociobiol. 70(11):1931–1939. 10.1007/s00265-016-2199-7

[CIT0036] Iglesias-Carrasco M , Zhang J, Noble DWA. 2024. Data from: Maternal investment and early thermal conditions affect performance and antipredator responses. Behav Ecol. 10.5061/dryad.vmcvdnd1c PMC11107847 38779594

[CIT0037] Johnson JM , Smaga CR, Bock SL, Parrott BB. 2023. Maternal provisioning interacts with incubation temperature to affect hatchling mercury exposure in an oviparous reptile. Biol Lett. 19(8):20230097. 10.1098/rsbl.2023.0097 37554010 PMC10410221

[CIT0038] Lancaster LT , McAdam AG, Sinervo B. 2010. Maternal adjustment of egg size organizes alternative escape behaviors, promoting adaptive phenotypic integration. Evolution. 64(6):1607–1621. 10.1111/j.1558-5646.2010.00941.x 20624182

[CIT0039] Langkilde T , Shine R. 2005. Different optimal offspring sizes for sons versus daughters may favor the evolution of temperature-dependent sex determination in viviparous lizards. Evolution. 59(10):2275–2280. 10.1554/05-239.1 16405171

[CIT0040] Leivesley JA , Rollinson N. 2021. Maternal provisioning and fluctuating thermal regimes enhance immune response in a reptile with temperature-dependent sex determination. J Exp Biol. 224(Pt 5):jeb237016. 10.1242/jeb.237016 33536300

[CIT0041] Lorioux S , Vaugoyeau M, DeNardo DF, Clobert J, Guillon M, Lourdais O. 2013. Stage dependence of phenotypical and phenological maternal effects: insight into squamate reptile reproductive strategies. Am Naturalist. 182(2):223–233. 10.1086/670809 23852356

[CIT0042] Marco A , Abella E, Martins S, López O, Patino-Martinez J. 2018. Female nesting behaviour affects hatchling survival and sex ratio in the loggerhead sea turtle: implications for conservation programmes. Ethol Ecol Evol. 30(2):141–155. 10.1080/03949370.2017.1330291

[CIT0043] Martin J , López P. 1999. When to come out from a refuge: risk sensitive and state-dependent decisions in an alpine lizard. Behav Ecol. 10(5):487–492. 10.1093/beheco/10.5.487

[CIT0044] McDonald S , Schwanz LE. 2018. Thermal parental effects on offspring behaviour and their fitness consequences. Anim Behav. 135:45–55. 10.1016/j.anbehav.2017.11.007

[CIT0045] Mcghee KE , Pintor LM, Suhr EL, Bell AM. 2012. Maternal exposure to predation risk decreases offspring antipredator behaviour and survival in threespined stickleback. Funct Ecol. 26(4):932–940. 10.1111/j.1365-2435.2012.02008.x 22962510 PMC3434968

[CIT0046] Monnahan CC , Thorson JT, Branch TA. 2017. Faster estimation of Bayesian models in ecology using Hamiltonian Monte Carlo. Methods Ecol Evol. 8(3):339–348. 10.1111/2041-210x.12681

[CIT0047] Noble DWA , Mcfarlane SE, Keogh JS, Whiting MJ. 2014. Maternal and additive genetic effects contribute to variation in offspring traits in a lizard. Behav Ecol. 25(3):633–640. 10.1093/beheco/aru032

[CIT0048] Noble DWA , Nakagawa S. 2021. Planned missing data designs and methods: options for strengthening inference, increasing research efficiency and improving animal welfare in ecological and evolutionary research. Evol Appl. 14(8):1958–1968. 10.1111/eva.13273 34429741 PMC8372070

[CIT0049] Noble DWA , Stenhouse V, Schwanz LE. 2018. Developmental temperatures and phenotypic plasticity in reptiles: a systematic review and meta-analysis. Biol Rev Camb Philos Soc. 93(1):72–97. 10.1111/brv.12333 28464349

[CIT0050] O’Dea RE , Noble DWA, Nakagawa S. 2022. Unifying individual differences in personality, predictability and plasticity: a practical guide. Methods Ecol Evol. 13(2):278–293. 10.1111/2041-210X.13755

[CIT0051] Oostra V , Saastamoinen M, Zwaan BJ, Wheat CW. 2018. Strong phenotypic plasticity limits potential for evolutionary responses to climate change. Nat Commun. 9(1):1–11. 10.1038/s41467-018-03384-9 29520061 PMC5843647

[CIT0052] Parmesan C. 2006. Ecological and evolutionary responses to recent climate change. Annu Rev Ecol Evol Syst. 37(1):637–669. 10.1146/annurev.ecolsys.37.091305.110100

[CIT0053] Pearson PR , Warner DA. 2016. Habitat- and season-specific temperatures affect phenotypic development of hatchling lizards. Biol Lett. 12(10):20160646. 10.1098/rsbl.2016.0646 28120809 PMC5095198

[CIT0054] Qualls FJ , Shine R. 1997. Geographic variation in “costs of reproduction” in the scincid lizard *Lampropholis guichenoti*. Funct Ecol. 11(6):757–763. 10.1046/j.1365-2435.1997.00150.x

[CIT0055] Qualls FJ , Shine R. 1998. Geographic variation in lizard phenotypes: importance of the incubation environment. Biol J Linn Soc. 64(4):477–491. 10.1111/j.1095-8312.1998.tb00345.x

[CIT0056] Räsänen K , Laurila A, Merilä J. 2005. Maternal investment in egg size: environment- and population-specific effects on offspring performance. Oecologia 142(4):546–553. 10.1007/s00442-004-1762-5 15688215

[CIT0057] Raynal RS , Noble DWA, Riley JL, Senior AM, Warner DA, While GM, Schwanz LE. 2022. Impact of fluctuating developmental temperatures on phenotypic traits in reptiles: a meta-analysis. J Exp Biol. 225(Suppl_1):jeb243369. 10.1242/jeb.243369 35258602

[CIT0058] Refsnider JM , Janzen FJ. 2012. Behavioural plasticity may compensate for climate change in a long-lived reptile with temperature-dependent sex determination. Biol Conserv. 152:90–95. 10.1016/j.biocon.2012.03.019

[CIT0059] Rosenfeld CS , Roberts RM. 2004. Maternal diet and other factors affecting offspring sex ratio: a review. Biol Reprod. 71(4):1063–1070. 10.1095/biolreprod.104.030890 15229140

[CIT0060] Rutstein AN , Gilbert L, Slater PJB, Graves JA. 2005. Sex-specific patterns of yolk androgen allocation depend on maternal diet in the zebra finch. Behav Ecol. 16(1):62–69. 10.1093/beheco/arh123

[CIT0061] Saaristo M , Brodin T, Balshine S, Bertram MG, Brooks BW, Ehlman SM, McCallum ES, Sih A, Sundin J, Wong BBM, et al. 2018. Direct and indirect effects of chemical contaminants on the behaviour, ecology and evolution of wildlife. Proc Biol Sci. 285(1885):20181297. 10.1098/rspb.2018.1297 30135169 PMC6125903

[CIT0062] Sanger TJ , Kyrkos J, Lachance DJ, Czesny B, Stroud JT. 2018. The effects of thermal stress on the early development of the lizard *Anolis sagrei*. J Exp Zool A Ecol Integr Physiol. 329(4-5):244–251. 10.1002/jez.2185 29938930

[CIT0063] Schwanz LE. 2016. Parental thermal environment alters offspring sex ratio and fitness in an oviparous lizard. J Exp Biol. 219(Pt 15):2349–2357. 10.1242/jeb.139972 27229475

[CIT0064] Schwanz LE , Janzen FJ. 2008. Climate change and temperature-dependent sex determination: can individual plasticity in nesting phenology prevent extreme sex ratios? Physiol Biochem Zool 81(6):826–834. 10.1086/590220 18831689

[CIT0065] Sharda S , Zuest T, Erb M, Taborsky B. 2021. Predator-induced maternal effects determine adaptive antipredator behaviors via egg composition. Proc Natl Acad Sci USA. 118(37):e2017063118. 10.1073/pnas.2017063118 34507981 PMC8449421

[CIT0066] Shine R , Downes SJ. 1999. Can pregnant lizards adjust their offspring phenotypes to environmental conditions? Oecologia 119(1):1–8. 10.1007/s004420050754 28308149

[CIT0067] Sinervo B. 1990. The evolution of maternal investment in lizards: an experimental and comparative analysis of egg size and its effects on offspring performance. Evolution. 44(2):279–294. 10.1111/j.1558-5646.1990.tb05198.x 28564384

[CIT0068] Sinervo B , Basolo A. 1996. Testing adaptation using phenotypic manipulations. In: Rose M, Lauder G, editors. Adaptation. CA: Academic. p. 149–185.

[CIT0069] Sinervo B , Doughty P, Huey RB, Zamudio K. 1992. Allometric engineering: a causal analysis of natural selection on offspring size. Science. 258(5090):1927–1930. 17836187 10.1126/science.258.5090.1927

[CIT0070] Singh SK , Das D, Rhen T. 2020. Embryonic temperature programs phenotype in reptiles. Front Physiol. 11:35. 10.3389/fphys.2020.00035 32082193 PMC7005678

[CIT0071] Ślipiński P , Trigos-Peral G, Maák I, Wojciechowska I, Witek M. 2021. The influence of age and development temperature on the temperature-related foraging risk of *Formica cinerea* ants. Behav Ecol Sociobiol. 75(7):107. 10.1007/s00265-021-03029-w

[CIT0072] Stan Development Team . 2020. RStan: The R interface to Stan. R package version 2.32. 6, https://mc-stan.org/

[CIT0073] Thompson MB , Speake BK, Russell KJ, McCartney RJ. 2001. Utilisation of lipids, protein, ions and energy during embryonic development of Australian oviparous skinks in the genus *Lampropholis*. Comp biochem physiol A Mol Integr Physiol. 129(2-3):313–326. 10.1016/s1095-6433(00)00349-4 11423304

[CIT0074] Tiatragul S , Kurniawan A, Kolbe JJ, Warner DA. 2017. Embryos of non-native anoles are robust to urban thermal environments. J Therm Biol. 65:119–124. 10.1016/j.jtherbio.2017.02.021 28343564

[CIT0075] Valenzuela N , Lance VA. 2004. Temperature-Dependent Sex Determination in Vertebrates. Washington DC: Smithsonian Books.

[CIT0076] Van Dyke JU , Griffith OW. 2018. Mechanisms of reproductive allocation as drivers of developmental plasticity in reptiles. J Exp Zool Part A: Ecol Integr Physiol. 329(6-7):275–286. 10.1002/jez.2165 29733527

[CIT0077] Walker WH , Meléndez-Fernández OH, Nelson RJ, Reiter RJ. 2019. Global climate change and invariable photoperiods: a mismatch that jeopardizes animal fitness. Ecol Evol. 9(17):10044–10054. 10.1002/ece3.5537 31534712 PMC6745832

[CIT0078] Warner DA , Andrews RM. 2002. Laboratory and field experiments identify sources of variation in phenotypes and survival of hatchling lizards. Biol J Linn Soc. 76(1):105–124. 10.1111/j.1095-8312.2002.tb01718.x

[CIT0079] Warner DA , Lovern MB. 2014. The maternal environment affects offspring viability via an indirect effect of yolk investment on offspring size. Physiol Biochem Zool 87(2):276–287. 10.1086/674454 24642545

[CIT0080] Warner DA , Lovern MB, Shine R. 2007. Maternal nutrition affects reproductive output and sex allocation in a lizard with environmental sex determination. Proc Biol Sci. 274(1611):883–890. 10.1098/rspb.2006.0105 17251109 PMC2093968

[CIT0081] Webb JK , Brown GP, Shine R. 2001. Body size, locomotor speed and antipredator behaviour in a tropical snake (*Tropidonophis mairii, colubridae*): the influence of incubation environments and genetic factors. Funct Ecol. 15(5):561–568. 10.1046/j.0269-8463.2001.00570.x

[CIT0082] Webb JK , Shine R, Christian KA. 2006. The adaptive significance of reptilian viviparity in the tropics: testing the maternal manipulation hypothesis. Evolution. 60(1):115–122. 10.1554/05-460.1 16568637

[CIT0083] While GM , Noble DWA, Uller T, Warner DA, Riley JL, Du WG, Schwanz LE. 2018. Patterns of developmental plasticity in response to incubation temperature in reptiles. J Exp Zool Part A: Ecol Integr Physiol. 329(4-5):162–176. 10.1002/jez.2181 29806741

[CIT0084] Wilson S , Swan G. 2021. A complete guide to reptiles of Australia (6th ed.). Sydney: Reed New Holland.

[CIT0085] Wolf JB , Wade MJ. 2009. What are maternal effects (and what are they not)? Philos Trans R Soc London Ser B. 364(1520):1107–1115. 10.1098/rstb.2008.0238 19324615 PMC2666680

[CIT0086] Yeh PJ , Price TD. 2004. Adaptive phenotypic plasticity and the successful colonization of a novel environment. Am Naturalist. 164(4):531–542. 10.1086/423825 15459883

[CIT0087] Zhang R , Wild K, Pottier P, Iglesias-Carrasco M, Nakagawa S, Noble D. 2023. Developmental environments do not affect thermal physiological traits in reptiles: an experimental test and meta-analysis. Biol Lett. 19:20230019. 10.1098/rsbl.2023.0019. 37161297 PMC10170202

