# Supplementary material for: Maternal investment and early thermal conditions affect performance and antipredator responses
Source: Behav Ecol. 2024 Apr 26;35(4):arae035. doi: 10.1093/beheco/arae035 (PMC11107847; doi:10.1093/beheco/arae035)
Supplement: arae035_suppl_Supplementary_Material [file arae035_suppl_supplementary_material.docx]

Supplementary material for

**Maternal resources and early thermal conditions affect performance and antipredator responses**

**Supplementary results**

**Table S1.** Percentage of the data missing for each behavioural trait. The large percentage observed for hiding time is the result of animals not leaving the shelter in the amount of time recorded (around 90 minutes after the simulated predatory attack).

| **Species** | **Hiding time** | **Time to activity** | **Distance moved** | **1m speed** | **Burst speed** |
| --- | --- | --- | --- | --- | --- |
| *L. delicata* | 44.71 | 27.06 | 1.18 | 0.39 | 0.39 |
| *L. guichenoti* | 22.87 | 8.14 | 1.16 | 0 | 0 |

**Table S2.** Repeatability of the three performance and antipredatory behaviour measures.

|  | ***L. delicata*** | | | ***L. guichenoti*** | | |
| --- | --- | --- | --- | --- | --- | --- |
|  | R | l | U | R | l | u |
| Hiding time | 0.2807 | 0.0222 | 0.5025 | 0.2391 | 0.0612 | 0.4072 |
| Time to activity | 0.0724 | 0.0001 | 0.2431 | 0.2315 | 0.0307 | 0.4093 |
| Distance moved | 0.2644 | 0.0406 | 0.4859 | 0.4824 | 0.3425 | 0.6092 |
| Running velocity 1m | 0.4580 | 0.3128 | 0.5893 | 0.5579 | 0.4339 | 0.6698 |
| Running velocity 25cm | 0.3440 | 0.2055 | 0.4812 | 0.6310 | 0.5223 | 0.7294 |

**Table S3.** Within-individual correlations between morphological traits estimated from Bayesian multivariate models for morphology in *Lampropholis delicata and L. guichenoti*.

| ***L. delicata*** |  |  |  |  |  |  |  |
| --- | --- | --- | --- | --- | --- | --- | --- |
| *Within-individual correlations in morphological traits* | | | |  |  |  |  |
|  | **Estimate** | **SE** | **l-95% CI** | **U-95% CI** | **Rhat** | **Bulk_ESS** | **Tail_ESS** |
| **rescor(SVL, Weight)** | **0.8** | **0.04** | **0.7** | **0.87** | **1** | **12001** | **10099** |
| **rescor(SVL, Tail length)** | **0.53** | **0.09** | **0.33** | **0.7** | **1** | **11828** | **10260** |
| **rescor(Weight, Tail length)** | **0.56** | **0.09** | **0.36** | **0.72** | **1** | **12109** | **10709** |
|  |  |  |  |  |  |  |  |
|  |  |  |  |  |  |  |  |
| ***L. guichenoti*** |  |  |  |  |  |  |  |
| *Within-individual correlations in morphological traits* | | | |  |  |  |  |
|  | **Estimate** | **Est.Error** | **l-95% CI** | **U-95% CI** | **Rhat** | **Bulk_ESS** | **Tail_ESS** |
| **rescor(SVL, Weight)** | **0.69** | **0.06** | **0.55** | **0.8** | **1** | **6372** | **7408** |
| **rescor(SVL, Tail length)** | **0.63** | **0.08** | **0.45** | **0.77** | **1** | **5176** | **6727** |
| **rescor(Weight, Tail length)** | **0.88** | **0.04** | **0.79** | **0.93** | **1** | **3383** | **6556** |

**Table** **S4**. Posterior means and 95% credible intervals for the interaction between temperature (Temp) and maternal investment (Invest) along with the main effects of temperature and maternal investment on behavioural and performance traits for *Lampropholis delicata* and *Lampropholis guichenoti*. Main effects are pooled posterior means over each level of second predictor variable. Estimates are from a Bayesian multivariate (multi-response) model controlling for SVL (Z-transformed).

| **Species** | **Trait** | **Term** | **Estimate** | **Q2.5** | **Q97.5** | **pMCMC** |
| --- | --- | --- | --- | --- | --- | --- |
| *L. delicata* | Time to activity (s) | **Interaction [(C23 - A23) - (C28 - A28)]** | **-1,003.752** | **-1,988.452** | **-33.590** | **0.044** |
|  |  | Temp (23-28) | -468.643 | -1,589.133 | 597.340 | 0.543 |
|  |  | Invest (C-A) | 122.220 | -1,014.488 | 1,158.751 | 0.873 |
|  | Hiding time (s) | *Interaction [(C23 - A23) - (C28 - A28)]* | *-896.713* | *-1,803.445* | *24.917* | *0.057* |
|  |  | Temp (23-28) | -423.236 | -1,444.311 | 558.994 | 0.544 |
|  |  | Invest (C-A) | 62.101 | -961.269 | 1,022.564 | 0.922 |
|  | Distance Moved (cm) | Interaction [(C23 - A23) - (C28 - A28)] | 54.939 | -174.283 | 295.328 | 0.642 |
|  |  | Temp (23-28) | 56.787 | -120.822 | 231.572 | 0.522 |
|  |  | Invest (C-A) | -35.953 | -206.360 | 140.925 | 0.668 |
|  | log 1m Speed (cm/s) | Interaction [(C23 - A23) - (C28 - A28)] | 0.312 | -0.071 | 0.692 | 0.109 |
|  |  | Temp (23-28) | -0.054 | -0.439 | 0.334 | 0.826 |
|  |  | Invest (C-A) | -0.267 | -0.646 | 0.109 | 0.198 |
|  | log Burst Speed (cm/s) | Interaction [(C23 - A23) - (C28 - A28)] | 0.184 | -0.206 | 0.577 | 0.352 |
|  |  | Temp (23-28) | -0.129 | -0.454 | 0.210 | 0.457 |
|  |  | Invest (C-A) | -0.098 | -0.423 | 0.223 | 0.566 |
| *L. guichenoti* | Time to activity (s) | Interaction [(C23 - A23) - (C28 - A28)] | -105.815 | -1,301.414 | 1,101.039 | 0.863 |
|  |  | Temp (23-28) | 43.518 | -893.814 | 998.022 | 0.935 |
|  |  | Invest (C-A) | 108.002 | -750.916 | 976.971 | 0.809 |
|  | Hiding time (s) | Interaction [(C23 - A23) - (C28 - A28)] | 298.455 | -574.277 | 1,159.178 | 0.499 |
|  |  | Temp (23-28) | -65.151 | -808.196 | 660.997 | 0.867 |
|  |  | Invest (C-A) | 49.173 | -628.295 | 718.879 | 0.884 |
|  | Distance Moved (cm) | Interaction [(C23 - A23) - (C28 - A28)] | 130.568 | -198.951 | 467.296 | 0.440 |
|  |  | Temp (23-28) | 8.658 | -287.891 | 299.148 | 0.943 |
|  |  | Invest (C-A) | -53.818 | -323.685 | 212.532 | 0.697 |
|  | log 1m Speed (cm/s) | Interaction [(C23 - A23) - (C28 - A28)] | -0.135 | -0.528 | 0.265 | 0.493 |
|  |  | Temp (23-28) | 0.024 | -0.307 | 0.358 | 0.887 |
|  |  | Invest (C-A) | -0.008 | -0.318 | 0.301 | 0.962 |
|  | log Burst Speed (cm/s) | **Interaction [(C23 - A23) - (C28 - A28)]** | **-0.413** | **-0.810** | **-0.006** | **0.046** |
|  |  | Temp (23-28) | -0.015 | -0.483 | 0.452 | 0.956 |
|  |  | Invest (C-A) | 0.008 | -0.432 | 0.455 | 0.991 |

**Table** **S5.** Posterior means and 95% credible intervals for the between and within-individual correlations among behavioural traits in *Lampropholis delicata* and *Lampropholis guichenoti*. Estimates are from a Bayesian multivariate (multi-response) model. Bold estimates indicate ones where the 95% credible interval does not overlap zero.

| **Species - level** | **Correlation** | **Estimate** | **2.5 % CI** | **97.5 % CI** |
| --- | --- | --- | --- | --- |
| *L. delicata* - Between Individual | cor(Time to Activity (s), Hiding Time(s)) | 0.29159827 | -0.60960494 | 0.868097048 |
|  | cor(Time to Activity (s), Distance Moved (cm)) | -0.13655106 | -0.72829302 | 0.620152880 |
|  | cor(Hiding Time(s), Distance Moved (cm)) | -0.13688198 | -0.70275908 | 0.579493448 |
|  | cor(Time to Activity (s), 1m Speed (cm/s)) | 0.27195884 | -0.38825792 | 0.776064363 |
|  | cor(Hiding Time(s), 1m Speed (cm/s)) | 0.15041240 | -0.46974706 | 0.688090745 |
|  | cor(Distance Moved (cm), 1m Speed (cm/s)) | 0.24238409 | -0.13979502 | 0.628382729 |
|  | cor(Time to Activity (s), Burst Speed (cm/s)) | 0.22520672 | -0.46104064 | 0.762376365 |
|  | cor(Hiding Time(s), Burst Speed (cm/s)) | 0.19915158 | -0.42866231 | 0.739702151 |
|  | cor(Distance Moved (cm), Burst Speed (cm/s)) | 0.26857069 | -0.12762202 | 0.664960881 |
|  | cor(1m Speed (cm/s), Burst Speed (cm/s)) | **0.82102166** | **0.62546459** | **0.952074963** |
| *L. delicata* - Within Individual | cor(Time to Activity (s), Hiding Time(s)) | **0.81659503** | **0.73344677** | **0.880468668** |
|  | cor(Time to Activity (s), Distance Moved (cm)) | -0.15311562 | -0.33138147 | 0.034234536 |
|  | cor(Hiding Time(s), Distance Moved (cm)) | -0.07303817 | -0.24215358 | 0.098905974 |
|  | cor(Time to Activity (s), 1m Speed (cm/s)) | 0.07619062 | -0.11077008 | 0.260270547 |
|  | cor(Hiding Time(s), 1m Speed (cm/s)) | 0.01950419 | -0.15776967 | 0.196633984 |
|  | cor(Distance Moved (cm), 1m Speed (cm/s)) | 0.01958401 | -0.13491722 | 0.169922742 |
|  | cor(Time to Activity (s), Burst Speed (cm/s)) | 0.02807736 | -0.18700479 | 0.239544933 |
|  | cor(Hiding Time(s), Burst Speed (cm/s)) | -0.04548645 | -0.24981026 | 0.159714536 |
|  | cor(Distance Moved (cm), Burst Speed (cm/s)) | 0.01922480 | -0.12981225 | 0.169160469 |
|  | cor(1m Speed (cm/s), Burst Speed (cm/s)) | **0.42413076** | **0.29703469** | **0.542793049** |
| *L. guichenoti* - Between Individual | cor(Time to Activity (s), Hiding Time(s)) | **0.90369565** | **0.77529782** | **0.978227159** |
|  | cor(Time to Activity (s), Distance Moved (cm)) | -0.45171409 | -0.69358613 | -0.177708817 |
|  | cor(Hiding Time(s), Distance Moved (cm)) | -0.29881986 | -0.57921282 | -0.002829739 |
|  | cor(Time to Activity (s), 1m Speed (cm/s)) | 0.24722197 | -0.03334559 | 0.511867690 |
|  | cor(Hiding Time(s), 1m Speed (cm/s)) | 0.19628177 | -0.08423812 | 0.469426798 |
|  | cor(Distance Moved (cm), 1m Speed (cm/s)) | -0.03441905 | -0.33088469 | 0.271057143 |
|  | cor(Time to Activity (s), Burst Speed (cm/s)) | **0.31610099** | **0.03933083** | **0.571799305** |
|  | cor(Hiding Time(s), Burst Speed (cm/s)) | 0.25109151 | -0.02586077 | 0.510175923 |
|  | cor(Distance Moved (cm), Burst Speed (cm/s)) | -0.15763758 | -0.43004251 | 0.126905402 |
|  | cor(1m Speed (cm/s), Burst Speed (cm/s)) | **0.95286559** | **0.89131640** | **0.989537957** |
| *L. guichenoti* - Within Individual | cor(Time to Activity (s), Hiding Time(s)) | **0.68721300** | **0.58149348** | **0.771981041** |
|  | cor(Time to Activity (s), Distance Moved (cm)) | -0.03812672 | -0.20063612 | 0.121080431 |
|  | cor(Hiding Time(s), Distance Moved (cm)) | 0.06659344 | -0.08967582 | 0.221268294 |
|  | cor(Time to Activity (s), 1m Speed (cm/s)) | -0.01538997 | -0.16973859 | 0.140063113 |
|  | cor(Hiding Time(s), 1m Speed (cm/s)) | -0.04596171 | -0.19493574 | 0.108994499 |
|  | cor(Distance Moved (cm), 1m Speed (cm/s)) | -0.03168564 | -0.17659253 | 0.116113479 |
|  | cor(Time to Activity (s), Burst Speed (cm/s)) | -0.04886429 | -0.21126303 | 0.117981964 |
|  | cor(Hiding Time(s), Burst Speed (cm/s)) | -0.02058361 | -0.17741319 | 0.138176209 |
|  | cor(Distance Moved (cm), Burst Speed (cm/s)) | 0.04028649 | -0.10947845 | 0.189531771 |
|  | cor(1m Speed (cm/s), Burst Speed (cm/s)) | **0.50825642** | **0.39813324** | **0.609206642** |
